# Comparison of scoliosis research society-22 between thoracic curve and lumbar/thoraco-lumbar curve in patients with adolescent idiopathic scoliosis

**DOI:** 10.1186/1748-7161-8-S1-P6

**Published:** 2013-06-03

**Authors:** H Iwakawa, J Takahashi, H Hirabayashi, N Ogihara, N Mukaiyama, S Kuraishi, M Shimizu, H Kato

**Affiliations:** 1 Dept. Orthop. Surg., Shinshu University Hospital, Matsumoto, Japan

## Background

The Scoliosis Research Society-22 (SRS-22) is a widely used instrument to measure clinical outcomes in patients with scoliosis. The comparison of domain scores in the SRS-22 between thoracic curve and lumbar/thoraco-lumbar curve has not been previously reported.

## Aim

The purpose of this study is to evaluate the comparison of domain scores in the SRS-22 between thoracic curve and lumbar/thoraco-lumbar curve in patients with adolescent idiopathic scoliosis (AIS).

## Methods

This is a cohort of 43 patients (all female) with AIS who underwent posterior spine fusion. The patients were divided into two group which were thoracic curve group (n=30, Lenke 1, 2, 3, 4) with a mean age of 14.6 years and a mean Cobb angle of 54° and lumbar/thoraco-lumbar group (n=14, Lenke 5, 6) with a mean age of 14.6 years and a mean Cobb angle of 56° Each was compared for preoperative SRS-22 domain scores.

## Results

In the thoracic curve group, the pre-operative mean values of appearance, activity, pain, mental, and sub-total were as follows: 2.8±0.5, 4.4±0.5, 4.1±0.4, 3.8±0.9, and 3.8±0.4, respectively. In the lumbar/thoraco-lumbar curve group, the preoperative mean values of appearance, activity, pain, mental, and sub-total were as follows: 2.7±0.5, 4.0±0.8, 3.8±0.9, 3.9±0.9, and 3.6±0.5, respectively. The pre-operative mean domain score of activity in the lumbar/thoraco-lumbar curve group was significantly smaller compare with that in the thoracic curve group (p=0.04).

## Conclusion

The pre-operative domain scores of appearance, pain and mental were almost the same; however, the pre-operative mean domain score of activity in the lumbar/thoraco-lumbar curve group was significantly smaller compared with that in the thoracic curve group.
